# Greening GenAI: sustainable solutions for higher education

**DOI:** 10.3389/frai.2026.1961109

**Published:** 2026-10-28

**Authors:** Björn B. de Koning, Marloes L. Nederhand

**Affiliations:** 1 Department of Psychology, Education and Child Studies, Erasmus School of Social and Behavioural Sciences, Erasmus University Rotterdam, Rotterdam, Netherlands; 2 Southampton Education School, University of Southampton, Southampton, United Kingdom

**Keywords:** environmental impact, generative AI, green AI, higher education, sustainability

## Abstract

Higher education institutions increasingly embrace and regulate Generative Artificial Intelligence (GenAI). While the cognitive, social, and ethical implications of GenAI are widely discussed in the current scientific discourse, the environmental disruption caused by large-scale GenAI use has remained largely ignored. As GenAI becomes integral to research and education, addressing its environmental impacts has become an increasingly urgent priority. In this Perspective, we argue that mitigating the environmental impact of GenAI requires more than advances in hardware and software efficiency; it also demands a stronger focus on user behavior as a key lever for reducing GenAI’s disruptive environmental impacts. This is particularly important given that every second, millions of prompts are submitted to GenAI systems in support of human learning, research, and knowledge acquisition. Optimizing how users interact with these systems offers scalable behavior-based strategies with the potential to substantially reduce their environmental impacts while requiring relatively modest changes in practice. We present a set of measures to promote the sustainable use of GenAI in higher education and scientific research.

## Introduction

Groundbreaking advancements in Generative Artificial Intelligence (GenAI) have transformed information processing to the point where it nearly matches human performance. Consequently, across industries and disciplines worldwide GenAI use has skyrocketed, with scholars, professionals, and students increasingly adopting GenAI in their daily routines (Heung and Chiu, 2025; Zheng, 2025). For example, already around 14% of biomedical articles until 2024 used GenAI (Kobak et al., 2025). This trend is expected to accelerate substantially in the coming years (Naddaf, 2025), with an expected tenfold GenAI usage in 2027 (De Vries, 2023). Hence, current global discourse and research on GenAI targets whether and how it should be integrated into universities and research practices, where its use is widespread and where students can be educated about its responsible use in relation to GenAI’s cognitive, social, and ethical dimensions (Jiao et al., 2025). However, an alarming issue remains largely absent in this debate: GenAI’s disruptive environmental impact.

This stands in contrast to the extensive body of research on the environmental costs of digital technology use, which highlights environmental impact across the technology life cycle, including energy use, emissions, infrastructure, and resource consumption (Malmodin et al., 2014), as well as indirect and rebound effects and broader environmental dimensions such as water, materials, and pollution (Roussilhe et al., 2023). With hundreds of millions of users worldwide, AI datacenters consume massive amounts of electricity and water, causing not only significant carbon emissions but also electronic waste. Vinuesa et al. indicate that due to AI’s reliance on non-renewable sources (e.g., natural gas and coal), AI can negatively impact 59 out of the 169 Sustainable Development Goals (SDGs) outlined by the United Nations (Vinuesa et al., 2020). Scientific research on GenAI’s environmental costs is only just emerging, as a significant obstacle for researchers lies in the current inability to reliably and validly quantify the exact impact of AI usage. Within the limited available scientific work, however, the findings are alarming. The estimated carbon footprint of AI systems is most recently estimated equivalent to that of the total emissions of New York City, the cumulative power demands approach that of a country like the United Kingdom, and the water footprint is estimated to be as large as the global annual bottled water consumption (De Vries-Gao, 2025). Paradoxically, while AI can potentially make meaningful contributions to enhance climate research and action, AI itself is one of the most energy-intensive digital technologies in use today (Tan et al., 2025; U.S. Congress, 2024).

Currently, the emerging scholarly work on GenAI’s environmental costs receives limited attention, and sustainability responsibilities are largely overlooked in studies and guidelines on how to use GenAI in scientific and educational contexts. Our central proposition in this Perspective is that if we allow and increasingly integrate GenAI usage at universities, in regulations and policies for both our scientific practices and education, we should urgently examine how to reach an optimal balance between the cognitive benefits of GenAI and its alarming environmental footprint. This aligns with literature on the Green AI perspective which advocates considering computational power alongside accuracy and other performance metrics when evaluating whether and how AI should be used [e.g., (Schwartz et al., 2020)]. Addressing this critical challenge requires an interdisciplinary approach but is currently predominantly confined to technical domains. Here, important efforts are made towards creating more efficient software (e.g., algorithms) and hardware (e.g., chips), as well as exploring more energy-efficient ways for training LLMs (Bolón-Canedo et al., 2024). On the other end of the technology, the (collective) user side, the disruptive environmental impact of GenAI has received hardly any attention. Research efforts (and practical applications) to study user behavior thus far primarily concentrate on identifying the cognitive challenges encountered when working with GenAI, like over-reliance on GenAI and reduced active processing, psycho-social benefits and drawbacks of using GenAI as a conversational partner, and ethical challenges for working with GenAI (Jiao et al., 2025). We therefore identify user behavior as an underexplored dimension of sustainable GenAI use and contend that changes in user behavior can deliver substantial gains both cognitively and environmentally. Evidence from other domains suggests that behavioral interventions can reduce resource consumption (Khanna et al., 2025), warranting investigation of their potential for GenAI. Importantly, this focus on user behavior does not imply that responsibility for sustainable GenAI is restricted to users alone. Rather, it is shared across users, universities, software and hardware developers, and infrastructure providers. Although much of GenAI’s environmental impact is determined by the architecture and infrastructure of the model and how it is institutionally used, insight into user behavior remains of vital importance. Given the growing numbers of users and how frequently they use GenAI, individual usage patterns can have cumulative environmental impacts. Moreover, understanding how users interact with GenAI can reveal opportunities to optimize system design and operation, potentially improving cognitive outcomes and environmental efficiency.

To pave the way forward, we discuss a research agenda on actionable user-oriented solutions to ensure cognitive benefits and reduced environmental costs. Building on Sustainable Interaction Design literature (Besana et al., 2024), we acknowledge that reducing the environmental impact of GenAI requires consideration of how, when, and for what purposes these technologies are used. Accordingly, we conceptualize efficient GenAI use not simply as minimizing technical computational resources, but as considering the resources required for human-AI interaction in relation to the quality and value of its outcome. This includes factors such as the number of prompts, AI model size, input and output length, computational demands, and whether the resulting educational or research outcome provides sufficient value to warrant the resources required, and the necessity of GenAI use for a given task.

### Greening GenAI prompt behavior

We consider prompt engineering, the strategic creation of prompts (input) to guide the behavior and desired outcomes (output) of LLMs (Garg et al., 2025), as a promising avenue to significantly influence the environmental footprint of GenAI, for better or worse depending on how it is used. In higher education, students generate a considerable number of prompts each time they use GenAI, with many using a ‘trial-and-error’ approach, prompting until they receive something valuable (Brocca and Garassino, 2026; Woo et al., 2025). This calls for prompt engineering education (Woo et al., 2025) while also suggesting that iterative prompting may lead to substantial computational power being heavily wasted during the process because people are not certain or thoughtful enough about what it is they request from GenAI. While on an individual level this might not be a major problem, when scaled globally in higher educational environments—where millions of students and researchers collectively craft billions of prompts—it becomes evident that prompting is an activity that currently imposes massive environmental costs. For example, Oviedo et al. (2026) estimate that serving 1 billion standard prompts per day would require approximately 0.7 GWh of electricity per day. Sustained over a year, this would amount to approximately 255.5 GWh of electricity, which is equivalent to the annual electricity consumption of approximately 100.000 Dutch households (Statistics Netherlands, 2024). Recent research has also begun to quantify the water footprint of AI computations. For example, a recent production-scale assessment of Google’s Gemini Apps estimates that a median text prompt consumes approximately 0.26 mL of water, alongside 0.24 Wh of electricity and 0.03 gCO₂e (Elsworth et al., 2025). Other research suggests substantially higher water consumption estimates for AI computations, highlighting the strong dependence of water use on model characteristics, data-center location, cooling systems, and the water intensity of electricity generation (Li et al., 2025). Together, this demonstrates that individual GenAI interactions have measurable resource requirements that can accumulate substantially when multiplied across large numbers of users and prompts.

Based on this, we advance the proposition that prompt engineering represents a critical leverage point for achieving substantial environmental gains when using GenAI. Currently available prompt engineering frameworks, such as CLEAR (Concise, Logical, Explicit, Adaptive, and Reflective) (Lo, 2023), are widely used but underexplored when it comes to balancing GenAI’s cognitive and environmental impacts (i.e., its efficiency). While providing helpful scaffolds, frameworks vary considerably and many have not been scientifically validated. Offering and scientifically examining the effects of prompt engineering trainings should become a top priority in universities if GenAI is allowed and embedded in the curriculum. This enables us to not only investigate if and how GenAI can enhance learning behaviors and study results, but importantly, facilitates more knowledge on how to train more efficient prompting. With ongoing advances in accurately measuring the exact energy consumption per GenAI interaction, such as the MELODI framework (Husom et al., 2024), it becomes increasingly feasible to determine the environmental impact of specific types of prompts. Ultimately, this can result in guidelines for prompt trainings focused on balancing the cognitive benefits with the environmental costs and on making deliberate choices for which activities using GenAI is worthwhile to institutionally embrace, and for which, and when, it is not.

### Safeguarding practice

Besides supporting users in efficient prompting, the specific platform they use for GenAI interactions and the amount of environmental impact it generates also serves as a source for solutions. Empirical benchmarking studies show that energy consumption varies across AI models and system configurations, with length and complexity of the output being an important determinant of energy use (Caravaca et al., 2025; Poddar et al., 2025). For example, a prompt in ChatGPT-4o consumes approximately 0.43 Wh of electricity, whereas a prompt processed by more reasoning-intensive models like DeepSeek-R1, can exceed 33 Wh per prompt. Moreover, energy consumption can be considerably higher for more demanding (e.g., reasoning-intensive) requests: prompts generating approximately 5.000 output tokens have been estimated at around 3.91 Wh, or roughly 13 times the energy of a standard prompt delivering a smaller output (Oviedo et al., 2026). These estimates illustrate how even relatively small per-prompt energy demands can translate into substantial aggregate energy consumption when GenAI is used at large scale.

As with any other technology, however, learning to work with GenAI does not come over night and inevitably involves making mistakes. This means that many useless prompts will be requested before users will master how to best and most efficiently interact with GenAI. We propose that in their way up to proficiency, significant environmental gains can be attained if users practice and train themselves in less computationally demanding and more environmentally ‘green’ GenAI environments. For example, at different universities, GenAI tools are created that work on a smaller scale than tools from big-tech companies like ChatGPT or Gemini. In the Netherlands, for example, EduGenAI is developed as a nationwide GenAI tool for higher education and secondary vocational education, in which one can choose the level of depth that is required from the LLM. Not every request requires a full search through all accessible information or an extensive response. Users themselves are able to choose an AI model appropriate to the complexity of their task, while setting boundaries on the depth of processing and output length, which may help reduce unnecessary computation and associated energy use (Poddar et al., 2025). Furthermore, the hardware of EduGenAI is partly hosted by the universities themselves, allowing for a clearer monitoring of the energy consumption that is caused by varying uses of GenAI. This can ultimately lead to better safeguards when students practice basic AI skills, with less advanced systems that require little computational power.

Safeguarding can also manifest in the way GenAI itself communicates. GenAI tools are characterized by their helpfulness, often offering follow-up questions (e.g., ‘Do you want me to use a more formal tone?’) after generating output. While this can potentially improve the quality of the final output up to a certain point, it poses a risk by encouraging ongoing interactions that lead to considerable negative environmental consequences without further improving the quality. This dynamic links to the persistent algorithms known from social media platforms, which tend to immerse users. When using GenAI, it is plausible that there may be an optimal point after which additional prompts contribute little, if at all, to the quality of the output. Understanding this optimum, also for different groups—i.e., a beginner may benefit more from GenAI’s generated follow-up questions, while an expert user may not need these—can benefit well-informed regulatory and institutional decisions of what kind of system scaffolding to use. This likely helps users minimize the environmental strain during their GenAI interactions almost automatically, while maintaining the quality of their work.

### Knowledge and awareness are key

Another solution-oriented step towards more responsible use of GenAI that we propose is to help scientists and students in not only learning *how* to use GenAI, but also in deciding *when* to use these tools. Interactions with GenAI tools such as ChatGPT and Copilot can include information-seeking behaviors that overlap with those in traditional search engines, including formulating, refining, and evaluating queries for responses. Research suggests that users can transfer and repurpose established search behaviors in GenAI searches, although the conversational nature of GenAI changes how these behaviors are enacted (Tibau et al., 2024; Kaiser et al., 2025). Despite these similarities in user search behavior, however, search engines operate fundamentally different from GenAI tools, both in their processes and the output they deliver. It is important for higher educational institutes that embrace GenAI to understand and inform its users of these differences, so that users can decide if they indeed need the computational power they initially requested.

In line with this, it is important to raise awareness among students and researchers about the environmental impact of the prompts they demand. Seemingly small changes, such as stopping to greet and to thank GenAI for its output, can on a large scale save computational power and resources—without causing any effects on the output that is generated. It should be noted that the most important issue is not politeness itself but unnecessary computation requested from GenAI tools. Adding a small number of courtesy tokens (i.e., small unit of text), such as “please” or “thank you,” to an otherwise necessary prompt is unlikely to result in a substantial environmental impact at the level of an individual interaction. However, each *additional* courtesy token requires the AI model to perform additional computations, and the cumulative effect can become meaningful when such unnecessary interactions are repeated at scale. For example, following Oviedo et al. (2026), 100 million ‘thank you’ prompts per day at 0.31 Wh per query would correspond to approximately 31 MWh of electricity per day, or around 11.3 GWh annually, which is equivalent to approximately the yearly electricity consumption of 4.400 Dutch households, which could have been easily prevented. Accordingly, users should not be discouraged from using polite language, but they can reduce environmental impact by avoiding unnecessary model calls, redundant iterations, and unnecessarily long generations. Raising awareness of these impacts may also become easier as GenAI tools are now being developed to show users metrics that provide information about the environmental footprint during their GenAI interactions. An important avenue for research in this area is twofold. On the one hand, a strong focus on better understanding and measuring the impact of specific interactions on GenAI’s energy consumption so that accurate information can be shown. And on the other hand, understanding how to best voice this knowledge to its users in a way that causes sustainable change—balancing individual responsibility with that of the institution (Chater and Loewenstein, 2023).

### Scientific debate and responsibility

The case we make for balancing the cognitive benefits and environmental costs pushes the educational and scientific debate around GenAI into new directions. At the discourse level, current debates about GenAI in higher education institutes largely center around its ethical concerns and questions of responsible use, often framed in terms of whether GenAI should be permitted. We move beyond questioning whether GenAI should be used as it is adapted on a large scale globally, but rather how we can effectively balance its environmental costs with its cognitive benefits. Being at the forefront of environmental research and global innovation, universities hold a unique responsibility in knowing and regulating their environmental (digital) footprint and are well-positioned to contribute to the discourse by urgently addressing the environmental costs with solid research and data. Furthermore, by employing millions of academics and educating millions of students worldwide, the impact universities can have through their regulations is immense. Universities can set norms, for example, for preregistering prompts used in research, and for more deliberate decisions in education for when usage of GenAI outweighs its costs because it benefits learning, and when it does not. Such regulations have a dual impact; they support researchers and teachers, who subsequently share their knowledge with students and societal stakeholders who can also benefit and further disseminate it, creating a snowball effect.

Together, we need to prioritize understanding how to increase awareness and skills in responsible and efficient use of GenAI. Understanding of the environmental footprint of combined with dedicated interventions to foster efficient and deliberate GenAI usage, can keep its costs within acceptable limits and can significantly benefit greener and more responsible use of GenAI.

## Conclusion

This perspective advances the debate on GenAI in higher education by foregrounding its largely overlooked disruptive environmental footprint and positioning user behavior as a key lever for change. Our call for user-centered strategies to green GenAI coincides with rapid advances in methods for quantifying the energy and resource demands (e.g., water use) of GenAI interactions (De Vries, 2023). These developments are making it increasingly possible to measure the environmental consequences of user behavior and to design and evaluate behavior-based interventions that promote more sustainable patterns of GenAI use.

Importantly, these behavioral interventions should not be considered in isolation given that they interact with GenAI model architectures, computational efficiency, and platform design thereby allowing the relative contributions of different stakeholders to be assessed. In other words, such interventions should be understood as complementary to, rather than substitutes for, improvements at the model, platform, and infrastructure levels. Higher education institutions can complement these efforts by creating an institutional environment that enables more sustainable GenAI use. This includes establishing clear criteria for the selection and procurement of GenAI tools, providing guidance of responsible GenAI use, and ensuring that sustainability is considered in research and innovation practices. GenAI tool developers and infrastructure providers, in turn, have a critical role in improving model and system efficiency, providing transparent environmental metrics, and designing interfaces that support more sustainable patterns of use.

Universities are uniquely positioned to act as living labs for such work because they combine intensive and varied use of GenAI with a strong educational and formative role. They therefore provide an environment in which sustainable patterns of GenAI use are not only studied and evaluated, but also actively developed through teaching, learning, and research. By incorporating environmental considerations into AI literacy and everyday academic practices, higher education can help students, teachers, and researchers make more informed decisions about when and how to use GenAI, while potentially contributing to the practices that students carry into their future professional lives. Essential to these endeavors is evaluating GenAI’s environmental impact in relation to the cognitive benefits it delivers. To illustrate, small increases in environmental costs may be justified by substantial cognitive gains, whereas substantial increases in environmental costs are difficult to justify when the resulting cognitive benefits remain marginal.

To conclude, greening GenAI use requires moving beyond purely technical optimization towards user-oriented and institutional interventions, including efficient prompt engineering, safeguarded practice in “greener” environments, and explicit decisions about when GenAI use is pedagogically warranted. Although this perspective article focuses on higher education, the proposed user-oriented directions for reducing GenAI’s environmental footprint may also be relevant beyond academia, including in workplaces, public-sector organizations, and other contexts where GenAI is increasingly adopted. Higher education can therefore serve as an important setting for experimentation and learning, while potentially serving as a catalyst for broader shifts towards more sustainable GenAI use.

## Data Availability

The original contributions presented in the study are included in the article/supplementary material, further inquiries can be directed to the corresponding author.

## References

[ref1] Besana N. Krogh P. G. Petersen M. G. Spallazzo D. (2024). Expanding the concept of sustainable interaction design: a systematic review. Sustainability 16:7486. doi: 10.3390/su16177486

[ref2] Bolón-Canedo V. Morán-Fernández L. Cancela B. Alonso-Betanzos A. (2024). A review of green artificial intelligence: Towards a more sustainable future. Neurocomputing 599:128096. doi: 10.1016/j.neucom.2024.128096

[ref3] Brocca N. Garassino D. (2026). LLMs in text linguistics teaching: An exploratory study with genAI novices in higher education. Comput. Educ. Open 11:100414. doi: 10.1016/j.caeo.2026.100414

[ref4] Caravaca F. Cuevas Á. Cuevas R. (2025). From prompts to power: measuring the energy. arXiv. doi: 10.48550/arXiv.2511.05597

[ref5] Chater N. Loewenstein G. (2023). The i-frame and the s-frame: how focusing on individual-level solutions has led behavioral public policy astray. Behav. Brain Sci. 46:e147. doi: 10.1017/S0140525X22002023, 36059098

[ref6] De Vries A. (2023). The growing energy footprint of artificial intelligence. Joule 7, 1161–1165. doi: 10.1016/j.joule.2023.09.004

[ref7] De Vries-Gao A. (2025). The carbon and water footprints of data centers and what this could mean for artificial intelligence. Patterns 7:101430. doi: 10.1016/j.patter.2025.101430 PMC12827721 41583976

[ref8] Elsworth C. Huang K. Patterson D. Schneider I. Sedivy R. Goodman S. . (2025). Measuring the environmental impact of delivering AI at Google Scale. arXiv. doi: 10.48550/arXiv.2508.15734

[ref9] Garg A. Soodhani K. N. Rajendran R. (2025). Enhancing data analysis and programming skills through structured prompt training: the impact of generative AI in engineering education. Comput. Educ. Artif. Intell. 8:100380. doi: 10.1016/j.caeai.2025.100380

[ref10] Heung Y. M. E. Chiu T. K. How ChatGPT impacts student engagement from a systematic review and meta-analysis study. Comput. Educ. 8,:100361 (2025). doi:10.1016/j.caeai.2025.100361

[ref11] Husom E. J. Goknil A. Shar L. K. Sen S. (2024). The price of prompting: profiling energy use in large language models inference. arXiv. doi: 10.48550/arXiv.2407.16893

[ref12] Jiao J. Afroogh S. Chen K. Atkinson D. Dhurandhar A. (2025). The global landscape of academic guidelines for generative AI and LLMs. Nat. Hum. Behav. 9, 638–642. doi: 10.1038/s41562-025-02124-6, 40033135

[ref13] Kaiser C. Kaiser J. Schallner R. Schneider S. (2025). Proceedings of the Extended Abstracts of the CHI Conference on Human Factors in Computing Systems. Association for Computing Machinery, 1–7.

[ref14] Khanna T. M. Danilenko D. Wang Q. Smith L. A. Bhumika T. V. Rai A. N. . (2025). Behavioral, information, and monetary interventions to reduce energy consumption in households: a living systematic review and network meta-analysis. Campbell Syst. Rev. 21:e70070. doi: 10.1002/cl2.70070 PMC11237337 38994501

[ref15] Kobak D. González-Márquez R. Horvát E.-A. Lause J. (2025). Delving into LLM-assisted writing in biomedical publications through excess vocabulary. Sci. Adv. 11:eadt3813. doi: 10.1126/sciadv.adt3813 PMC12219543 40601754

[ref16] Li N. Li H. Duan K. Tao W.-Q. (2025). Evaluation of the cooling effectiveness of air-cooled data centers by energy diagram. Appl. Energy 382:125215. doi: 10.1016/j.apenergy.2024.125215

[ref17] Lo L. S. (2023). The CLEAR path: a framework for enhancing information literacy through prompt engineering. J. Acad. Librarian. 49:102720. doi: 10.1016/j.acalib.2023.102720, 42574925

[ref18] Malmodin J. Moberg Å. Lundén D. Finnveden G. Lövehagen N. (2014). Greenhouse Gas Emissions and Operational Electricity Use in the ICT and Entertainment & Media Sectors. J. Ind. Ecol. 18, 398–411. doi: 10.1111/j.1530-9290.2010.00278.x

[ref19] Naddaf M. (2025). How are researchers using AI? Survey reveals pros and cons for science. Nature. doi: 10.1038/d41586-025-00343-5 39905251

[ref20] Oviedo F. Kazhamiaka F. Choukse E. Kim A. Luers A. Nakagawa M. . (2026). Energy use of AI inference, efficiency pathways, and test-time scaling. Joule 10:102430. doi: 10.1016/j.joule.2026.102430

[ref21] Poddar S. Koley P. Misra J. Ganguly N. Ghosh S. (2025). Proceedings of the 2025 Conference of the Nations of the Americas Chapter of the Association for Computational Linguistics: Human Language Technologies (Volume 1: Long Papers). Association for Computational Linguistics, 12688–12704.

[ref22] Roussilhe G. Ligozat A.-L. Quinton S. (2023). A long road ahead: a review of the state of knowledge of the environmental effects of digitization. Curr. Opin. Environ. Sustain. 62:101296. doi: 10.1016/j.cosust.2023.101296

[ref23] Schwartz R. Dodge J. Smith N. A. Etzioni O. (2020). Green AI. Commun. ACM 63, 54–63. doi: 10.1145/3381831

[ref24] Statistics Netherlands. (2024). Nearly a quarter of Dutch Population use AI Programs such as ChatGPT. Available online at: https://www.cbs.nl/en-gb/news/2024/36/nearly-a-quarter-of-dutch-population-use-ai-programs-such-as-chatgpt

[ref25] Tan X. Peng Z. Cheng Y. Wang Y. Chao Q. Huang X. . (2025). Leveraging artificial intelligence for research and action on climate change: opportunities, challenges, and future directions. Sci. Bull. 17, 2886–2893. doi: 10.1016/j.scib.2025.06.035 40651897

[ref26] Tibau M. Siqueira S. W. M. Nunes B. P. (2024). ChatGPT for chatting and searching: Repurposing search behavior. Libr. Inf. Sci. Res. 46:101331. doi: 10.1016/j.lisr.2024.101331

[ref27] U.S. Congress. (2024). S.3732—Artificial Intelligence Environmental Impacts act. Available online at: https://www.congress.gov/bill/118th-congress/senate-bill/3732/text (Accessed July 15, 2026).

[ref28] Vinuesa R. Azizpour H. Leite I. Balaam M. Dignum V. Domisch S. . (2020). The role of artificial intelligence in achieving the sustainable development goals. Nat. Commun. 11:233. doi: 10.1038/s41467-019-14108-y, 31932590 PMC6957485

[ref29] Woo D. J. Guo K. Susanto H. (2025). EFL secondary students’ use of ChatGPT for writing task completion pathways. J. Educ. Res. 118, 596–609. doi: 10.1080/00220671.2025.2510382

[ref30] Zheng Z. (2025). The future of reviews writing in the AI era. Nat. Rev. Chem. 9, 495–496. doi: 10.1038/s41570-025-00738-y 40596717

